# Transcriptomic Analysis of the Photosynthetic, Respiration, and Aerenchyma Adaptation Strategies in Bermudagrass (*Cynodon dactylon*) under Different Submergence Stress

**DOI:** 10.3390/ijms22157905

**Published:** 2021-07-23

**Authors:** Zhongxun Yuan, Xilu Ni, Muhammad Arif, Zhi Dong, Limiao Zhang, Xue Tan, Jiajia Li, Changxiao Li

**Affiliations:** 1 Key Laboratory of Eco-Environments in the Three Gorges Reservoir Region (Ministry of Education), Chongqing Key Laboratory of Plant Resource Conservation and Germplasm Innovation, College of Life Sciences, Southwest University, Chongqing 400715, China; yuanzhongxun@email.swu.edu.cn (Z.Y.); muhammadarif@swu.edu.cn (M.A.); zhidong@email.swu.edu.cn (Z.D.); lmzhang@email.swu.edu.cn (L.Z.); t12345x@email.swu.edu.cn (X.T.); ljj133888@email.swu.edu.cn (J.L.); 2 Breeding Base for State Key Laboratory of Land Degradation and Ecological Restoration of North-Western China, Key Lab for Restoration and Reconstruction of Degraded Ecosystem in North-Western China (Ministry of Education), Ningxia University, Yinchuan 750021, China; nixilu@nxu.edu.cn

**Keywords:** bermudagrass, transcriptomic analysis, submergence stress, differentially expressed genes, photosynthesis, energy metabolism, aerenchyma

## Abstract

Submergence impedes photosynthesis and respiration but facilitates aerenchyma formation in bermudagrass. Still, the regulatory genes underlying these physiological responses are unclear in the literature. To identify differentially expressed genes (DEGs) related to these physiological mechanisms, we studied the expression of DEGs in aboveground and underground tissues of bermudagrass after a 7 d treatment under control (CK), shallow submergence (SS), and deep submergence (DS). Results show that compared with CK, 12276 and 12559 DEGs were identified under SS and DS, respectively. Among them, the DEGs closely related to the metabolism of chlorophyll biosynthesis, light-harvesting, protein complex, and carbon fixation were down-regulated in SS and DS. Meanwhile, a large number of DEGs involved in starch and sucrose hydrolase activities, glycolysis/gluconeogenesis, tricarboxylic acid (TCA) cycle, and oxidative phosphorylation were down-regulated in aboveground tissues of bermudagrass in SS and DS. Whereas in underground tissues of bermudagrass these DEGs were all up-regulated under SS, only beta-fructofuranosidase and α-amylase related genes were up-regulated under DS. In addition, we found that DEGs associated with ethylene signaling, Ca^2+^-ROS signaling, and cell wall modification were also up-regulated during aerenchyma formation in underground tissues of bermudagrass under SS and DS. These results provide the basis for further exploration of the regulatory and functional genes related to the adaptability of bermudagrass to submergence.

## 1. Introduction

Climate change increases extreme rainfall and flood events, which impact the vegetation cover globally. Submergence stress has become one of the most significant global problems for vegetation deterioration. It has hampered social and economic development and has posed an increasingly severe threat to global ecological security. Many parts of the world are experiencing various degrees of submergence, including the water-level-fluctuation zones formed after the construction of artificial dams [1,2]. The Three Gorges Dam Reservoir (TGDR) is the world’s largest power station, and the water flow is controlled by the “Winter Storage Summer Drainage” operating mode, causing the annual water level of the reservoir to fluctuate between 145 m and 175 m above sea level (a.s.l.) [3,4,5]. Thus, a hydro-fluctuation belt was formed, stretching a length of 2000 km, with a vertical drop of 30 m over an area of 400 km^2^ [6]. The significant change in the growing environment and the frequent disturbance of anti-seasonal flooding resulted in a severe impact on native plants [5,7], causing a series of ecological and environmental problems in the reservoir area. Therefore, it is necessary to carry out studies on the mechanism of submergence-tolerant plants for ecosystem restoration of the hydro-fluctuation belt [8].

Bermudagrass (*Cynodon dactylon*), a warm-season C_4_ perennial grass, is widely distributed worldwide as turfgrass and can grow in various habitats, including drought, waterlogging, heat, high salinity, and so on. Moreover, bermudagrass is also the dominant and pioneer species in the riparian zone of the TGDR. It has a strong tolerance to submergence, and the survival rate is still 100% after submergence at a depth of 25 m for 216 days [6]. More importantly, its developed root systems and creeping stems play important roles in controlling soil erosion and stabilizing soil [9,10,11], and are thus able to effectively improve the local ecological environment in the riparian zone of the TGDR [12,13].

Previous studies on the submergence mechanism of bermudagrass mainly focused on physiological and biochemical responses in recent years. However, little information is available on the molecular mechanism of the photosynthetic response of bermudagrass to submergence. Photosynthesis is the most important metabolic activity of plants and the leading way for plants to store nutrients, which plays an important role in plant growth and development [14]. In most plants, sucrose is the end product of photosynthesis that can be translocated from photosynthetic tissue (source) to heterotrophic tissue (sink), and the hexoses produced by sucrose metabolism can generate energy for plant growth, development, and defense [15]. Previous investigations found that the net photosynthetic rate (*P*_n_), stomatal conductance (*G*_s_), transpiration rate (*T*_r_), and stomatal limitation (*L*_s_) of bermudagrass in waterlogging (submergence about 5 cm above the soil surface) were significantly lower compared with that in control [16]. Li and Li (2018) found that the soluble sugar content in the stem of bermudagrass was also significantly decreased under shallow submergence (above the soil 10 cm) and under deep submergence (above the soil 2 m) [17], whereas the soluble sugar content in the root of bermudagrass increased under shallow submergence and decreased under deep submergence. Meanwhile, due to the low-oxygen content and slow diffusion rate in the flooding environment, aerobic respiration of plants becomes limited [18,19,20], which results in a limited supply of energy for nutrient uptake and transport [21]. A former study documented that ethanol dehydrogenase activity in bermudagrass roots increased under submergence and increased submergence depth [22].

However, changing internal aeration conditions via aerenchyma formation is important for plants to survive in a flooding environment. In addition to aiding internal gas transport from aboveground to underground, the aerenchyma also reduces respiratory consumption and nutrient requirements [23,24]. Our previous research observed the root aerenchyma formation process through microstructure and ultrastructure studies and found that bermudagrass roots formed schizo-lysigenous aerenchyma even in an anaerobic environment (data not shown). Meanwhile, flooding further boosted the formation of the aerenchyma of bermudagrass. Aerenchyma is the main physiological and morphological response of plants to flooding stress, which improves the plant tolerance to flooding [25,26]. Some wetland plants (e.g., *Oryza sativa* and *Juncus effuses*) constitutively form aerenchyma in root under aerobic conditions and further enhance their formation under submergence or oxygen-deficient conditions [27,28]. Evans (2004) [29] proposed that cell death in the root cortex can be divided into five stages: (1) perceiving hypoxia and initiating ethylene biosynthesis; (2) perceiving ethylene signal; (3) initiating cell death; (4) increasing hydrolase activities; and (5) degrading cell wall [28,30]. However, the initiation and regulation of programmed cell death (PCD) is performed by an intricate signal network, and its related signal molecules include ethylene, calcium, and reactive oxygen species (ROS) [30].

So far, there is still a lack of deep understanding about why the bermudagrass could tolerate such extremely adverse submergence conditions, especially from the perspective of molecular studies. At present, the research on the adaptive molecular mechanism of bermudagrass at home and abroad has focused on salt tolerance, drought tolerance, and cold tolerance [31,32,33,34], but little has focused on submergence tolerance. In particular, due to the unclear genetic background of bermudagrass, studies on the effects of submergence on bermudagrass and bermudagrass adaptation mechanisms mainly focused on the physiology and biochemistry aspects; few studies focused on the transcriptome level. However, a couple of former studies documented the changes in differentially expressed genes (DEGs) in the stem of bermudagrass under flooding (flooding 30 cm) and control conditions [35,36]. They preliminarily analyzed the functional annotation and pathway enrichment of DEGs. However, given the distinct environmental constraints on aboveground and underground tissues under different submergence conditions, each organ might also perform different responses. Furthermore, the response and adaptability of plants to submergence are complex processes involving the co-participation and mutual coordination of many genes in multiple signaling pathways. Thus, it is of great importance to explore the effects of submergence on aboveground and underground tissues of bermudagrass and its adaptive mechanisms of multiple dynamic signaling pathways under different submergences.

Recently, with the rapid development of high-throughput sequencing technology, transcriptome research has provided extensive insights into the molecular processes by which plants respond to abiotic stresses [37]. To fill this knowledge gap, we studied the gene expression profiles in aboveground and underground tissues of bermudagrass under submergence and control conditions, respectively, and identified genes exhibiting significant transcriptional changes in bermudagrass, mainly through focusing on photosynthesis and metabolic pathways and aerenchyma formation. Simultaneously, the function of these DEGs was further analyzed by signaling pathways. By better understanding the regulatory genes of these adaptive mechanisms, it will be helpful to unearth related genes for flood tolerance, improve the submergence tolerance of bermudagrass, and provide references for subsequent screening and breeding of flood tolerant plants [18,35,36].

## 2. Results

### 2.1. Sequencing Quality Evaluation and Assembly

After a series of sequencing quality evaluations, a total of 44.38 Gb of clean data was obtained. The GC content of each sample was all higher than 52.71%, and the Q30 (base recognition accuracy was 99.9%) was greater than or equal to 88.32%. This indicated that the sequencing results and quality were sufficient for further analysis. Sequence alignment was conducted between clean data of each sample and the transcript or unigene library assembled. It was found that the mapped ratio was greater than or equal to 72.26% (Table 1).

### 2.2. Identification of DEGs under Different Water Regimes

**Figure 1:**
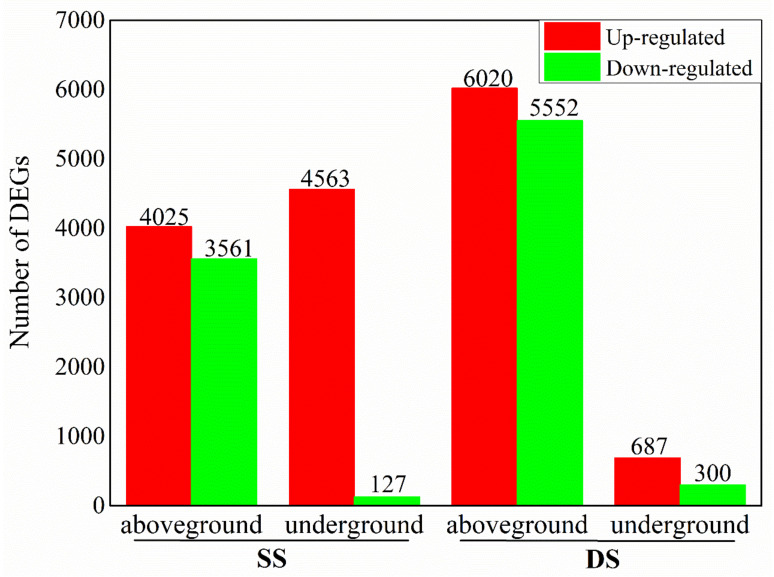
The number of differentially expressed genes (DEGs) in aboveground and underground tissues of bermudagrass under shallow submergence (SS) and deep submergence (DS).

The difference in gene expression was analyzed by the comparisons of control plants and plants under submergence, respectively. The results show that after treatment for 7 d under SS, 7586 and 4690 DEGs were identified in aboveground and underground tissues of bermudagrass. Among these DEGs, 4025 and 4563 DEGs were up-regulated, including 736 and 1686 DEGs that were expressed in aboveground and underground tissues of bermudagrass under shallow submergence (SS), respectively, but almost none in control (CK). The up-regulated expression of genes in aboveground and underground tissues of bermudagrass under SS was 2.04–14.71 (log_2_ FC) and 2.20–13.02 (log_2_ FC) fold change, respectively, where log_2_ FC represents log_2_ fold change. The amount of down-regulated DEGs was much less than that of up-regulated DEGs (Figure 1). A total of 3561 and 127 DEGs were down-regulated, of which 900 and 34 DEGs expressed in aboveground and underground parts of bermudagrass under CK, respectively, but almost none in SS. The down-regulated expression of genes in aboveground and underground parts of bermudagrass in SS was 2.05–15.01 (log_2_ FC) and 2.19–13.39 (log_2_ FC) fold change, respectively.

While treated for 7d under deep submergence (DS), 11572 and 987 DEGs were identified in aboveground and underground tissues of bermudagrass. Among these DEGs, 6020 and 687 DEGs were up-regulated, including 955 and 154 DEGs that expressed in aboveground and underground tissues of bermudagrass under DS, respectively, but almost none in CK. The up-regulated expression of genes in aboveground and underground tissues of bermudagrass under DS was 1.95–14.53 (log_2_ FC) and 2.46–13.93 (log_2_ FC) fold change, respectively. Likewise, the amount of down-regulated DEGs was also much less than that of up-regulated DEGs (Figure 1). A total of 5552 and 300 DEGs were down-regulated, of which 1269 and 91 DEGs were expressed in aboveground and underground parts of bermudagrass under CK, respectively, but almost none in DS. The down-regulated expression of genes in aboveground and underground tissues of bermudagrass in DS was 1.95–15.14 (log_2_ FC) and 2.49–13.35 (log_2_ FC) fold change, respectively.

### 2.3. DEGs Related to Photosynthesis

**Figure 2:**
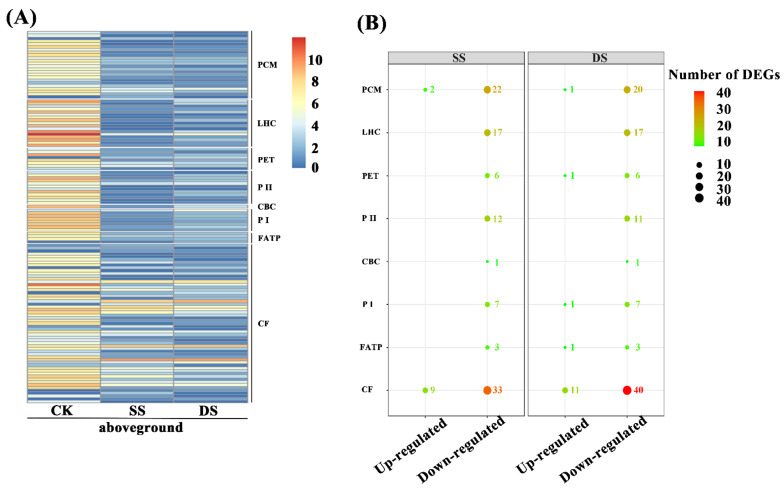
The expression of differentially expressed genes (DEGs) related to photosynthesis in aboveground tissues of bermudagrass. (**A**) Heatmap of DEGs expression profiles under control (CK), shallow submergence (SS), and deep submergence (DS). The *x* axis represents different treatments. The *y* axis represents the value of relative expression level (log_2_ (FPKM + 1)). (**B**) The number of DEGs expression under SS and DS. Point size correlates with the number of DEGs. FPKM: fragments per kilobase of transcript per million mapped reads; PCM: porphyrin and chlorophyll metabolism; LHC: light-harvesting chlorophyll protein complex; PET: photosynthethic electron transport; P II: photosystem II; CBC: cytochrome b6/f complex; P I: photosystem I; FATP: F-type ATPase; CF: carbon fixation.

The DEGs in four metabolic pathways closely associated with photosynthesis were analyzed, including porphyrin and chlorophyll metabolism, photosynthesis, light-harvesting chlorophyll protein complex, and carbon fixation. After 7 d water treatment, the expression levels of most of the DEGs related to photosynthesis in SS and DS were remarkably lower than in CK, as shown by the heatmap (Figure 2A), and 112 and 120 DEGs related to photosynthesis were identified in SS and DS, respectively (Figure 2B). Among these DEGs, 101 in SS and 105 DEGs in DS were markedly down-regulated. Totally, 22, 17, 6, 12, 1, 7, 3, and 33 down-regulated DEGs under SS and 20, 17, 6, 11, 1, 7, 3, and 40 down-regulated DEGs under DS were related to the porphyrin and chlorophyll metabolism, light-harvesting chlorophyll protein complex, photosynthethic electron transport, photosystem II, cytochrome b6/f complex, photosystem I, F-type ATPase, and carbon fixation, respectively (Figure 2B). Furthermore, the amount of up-regulated DEGs was much less than that of down-regulated DEGs, about which there were only 11 and 15 up-regulated DEGs in SS and DS, respectively.

**Figure 3:**
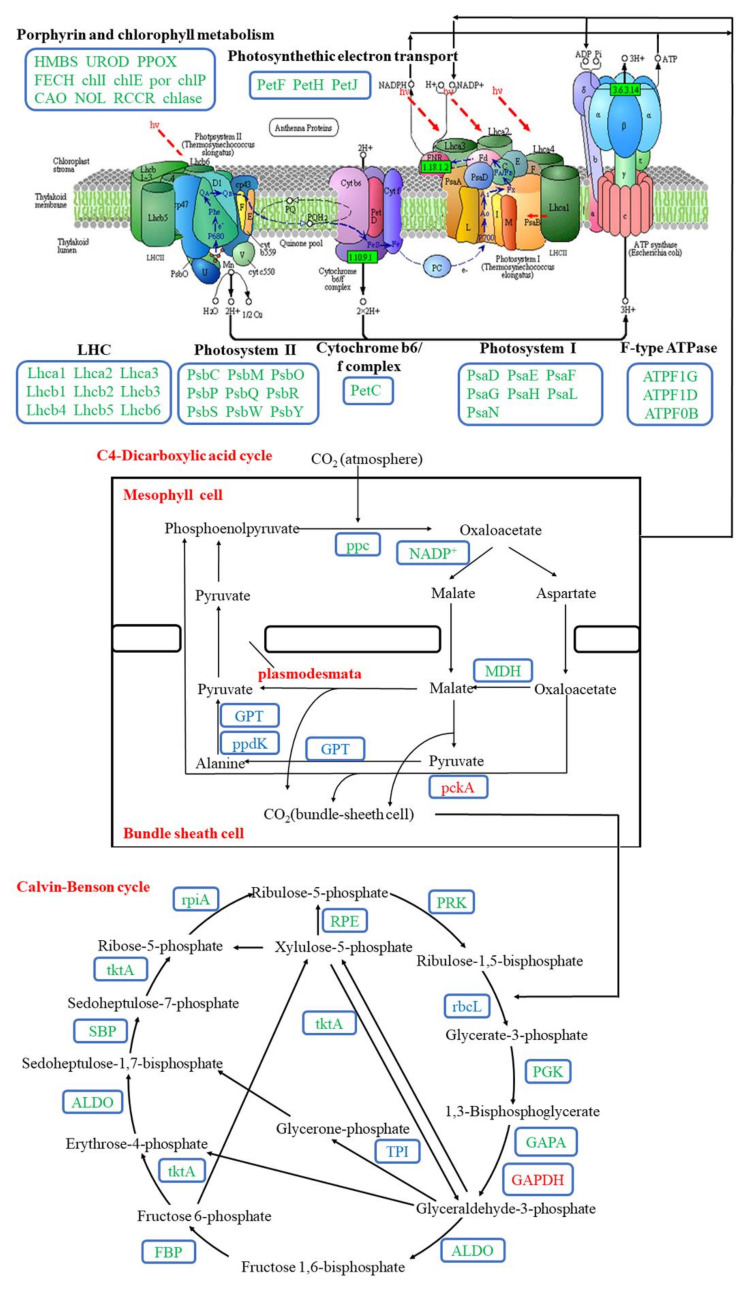
The co-expression of differentially expressed genes (DEGs) related to photosynthetic pathways in aboveground tissues of bermudagrass under shallow submergence (SS) and deep submergence (DS). Red letters represent up-regulated expression; green letters represent down-regulated expression; blue letters represent both up-regulated and down-regulated expression. HMBS: hydroxymethylbilane synthase; UROD: uroporphyrinogen decarboxylase; PPOX: protoporphyrinogen/coproporphyrinogen III oxidase; FECH: protoporphyrin/coproporphyrin ferrochelatase; chlI: magnesium chelatase subunit I; chlE: magnesium-protoporphyrin IX monomethyl ester (oxidative) cyclase; por: protochlorophyllide reductase; chlP: geranylgeranyl diphosphate/geranylgeranyl-bacteriochlorophyllide a reductase; CAO: chlorophyllide a oxygenase; NOL: chlorophyll (ide) b-reductase; RCCR: red chlorophyll catabolite reductase; chlase: chlorophyllase; Lhca1–3: light-harvesting complex I chlorophyll a/b binding protein 1–3; Lhcb1–6: light-harvesting complex II chlorophyll a/b binding protein 1–6; PetF: ferredoxin; PetH: ferredoxin-NADP^+^ reductase; PetJ: cytochrome c6; PsbC: photosystem II CP43 chlorophyll apoprotein; PsbM: photosystem II PsbM protein; PsbO: photosystem II oxygen-evolving enhancer protein 1; PsbP: photosystem II oxygen-evolving enhancer protein 2; PsbQ: photosystem II oxygen-evolving enhancer protein 3; PsbR: photosystem II 10kDa protein; PsbS: photosystem II 22kDa protein; PsbW: photosystem II PsbW protein; PsbY: photosystem II PsbY protein; PetC: cytochrome b6/f complex iron-sulfur subunit; PsaD, E, F, G, H, L: photosystem I subunit II, IV, III, V, VI, XI; PsaN: photosystem I subunit PsaN; ATPF0B, 1D, 1G: F-type H^+^-transporting ATPase subunit b, delta, gamma; ppc: phosphoenolpyruvate carboxylase; NADP^+^: malate dehydrogenase (NADP^+^); MDH: malate dehydrogenase; GPT: alanine transaminase; ppdK: pyruvate, orthophosphate dikinase; pckA: phosphoenolpyruvate carboxykinase; rpiA: ribose 5-phosphate isomerase A; PRK: phosphorribulokinase; rbcL: ribulose-bisphosphate carboxylase large chain; PGK: phosphoglycerate kinase; GAPA: glyceraldehyde-3-phosphate dehydrogenase (NADP^+^) (phosphorylating); GAPDH: glyceraldehyde 3-phosphate dehydrogenase; ALDO: fructose-bisphosphate aldolase, class I; FBP: fructose-1,6-bisphosphatase I; tktA: transketolase; SBP: sedoheptulose-bisphosphatase; RPE: ribulose-phosphate 3-epimerase; TPI: triosephosphate isomerase (TIM).

Moreover, we further summarized the co-expression of down-regulated DEGs under SS and DS, which were mainly divided into photosynthesis light system apparatus and carbon fixation (Figure 3). The expression level of genes coding several major protein complexes in photosynthesis light system apparatus were markedly down-regulated under flooding conditions, including genes related to porphyrin and chlorophyll (such as *HMBS*, *UROD*, *PPOX*, *FECH*, *chlI*, *chlE*, *por*, *chlP*, *CAO*, *NOL*, *RCCR*, and *chlase*), light-harvesting chlorophyll protein complex (*Lhca1*, *Lhca2*, *Lhca3*, *Lhcb1*, *Lhcb2*, *Lhcb3*, *Lhcb4*, *Lhcb5*, and *Lhcb6*), photosynthethic electron transport (*PetF*, *PetH*, and *PetJ*), photosystem II (such as *PsbC*, *PsbM*, *PsbO*, *PsbP*, *PsbQ*, *PsbR*, *PsbS*, *PsbW*, and *PsbY*), cytochrome b6/f complex (*PetC*), photosystem I (*PsaD*, *PsaE*, *PsaF*, *PsaG*, *PsaH*, *PsaL*, and *PsaN*), and F-type ATPase (*ATPF1G*, *ATPF1D*, and *ATPF0B*) (Figure 3). Furthermore, the expression level of genes coding some proteins (enzymes) in carbon fixation was also down-regulated, including *ppc*, *NADP^+^*, *MDH*, *PRK*, *PGK*, *GAPA*, *ALDO*, *FBP*, *tktA*, *SBP*, *rpiA*, and *RPE* (Figure 3).

### 2.4. DEGs Related to Energy Metabolism

**Figure 4:**
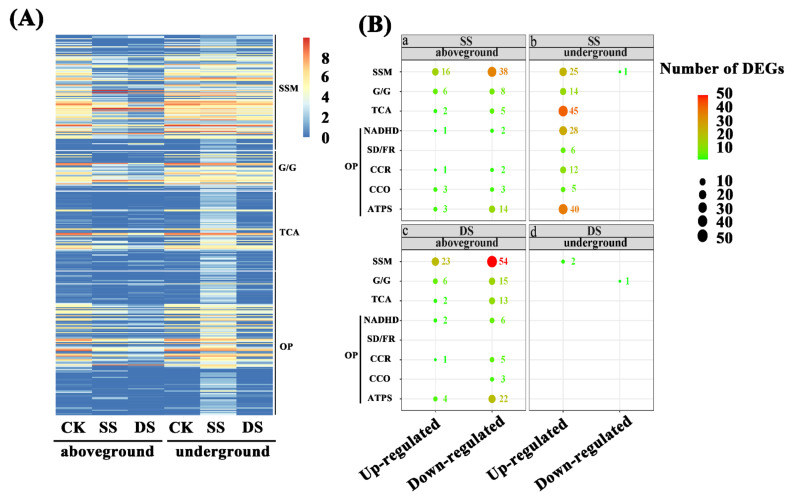
Differentially expressed genes (DEGs) related to energy metabolism in aboveground and underground tissues of bermudagrass. (**A**) Heatmap of DEGs expression profiles under control (CK), shallow submergence (SS), and deep submergence (DS). The *x* axis represents different treatments. The *y* axis represents the value of relative expression level (log_2_ (FPKM + 1)). (**B**) The number of DEGs expression under SS and DS. Point size correlates with the number of DEGs. FPKM: fragments per kilobase of transcript per million mapped reads; SSM: starch and sucrose metabolism; G/G: glycolysis/gluconeogenesis; TCA: TCA cycle; NADHD: NADH dehydrogenase; SD/FR: succinate dehydrogenase/fumarate reductase; CCR: cytochrome c reductase; CCO: cytochrome c oxidase; ATPS: ATP synthase; OP: oxidative phosphorylation.

We then analyzed four metabolic pathways closely related to energy metabolism, including glycolysis/gluconeogenesis, starch and sucrose metabolism, tricarboxylic acid (TCA) cycle, and oxidative phosphorylation. The oxidative phosphorylation pathway mainly included NADH dehydrogenase, cytochrome c reductase, cytochrome c oxidase, and adenosine triphosphate (ATP) synthase. To comprehensively provide the DEGs expression profile related to energy metabolism in aboveground and underground parts of bermudagrass under different water treatments, we observed the DEGs expression in the four metabolic pathways based on the expression models of log_2_ (FPKM + 1) (Figure 4A). After bermudagrass was treated for 7 d, 104 and 156 DEGs related to energy metabolism were observed in aboveground tissues of bermudagrass under SS and DS, respectively (Figure 4Ba,c). The amount of down-regulated DEGs (72 and 118 in total) was larger than that of the up-regulated DEGs (32 and 38 in total) under SS and DS, respectively. In total, 38, 8, 5, and 21 down-regulated DEGs and 16, 6, 2, and 8 up-regulated DEGs were involved in starch and sucrose metabolism, glycolysis/gluconeogenesis, TCA cycle, and oxidative phosphorylation, respectively, under SS. Meanwhile, 54, 15, 13, and 36 down-regulated DEGs and 23, 6, 2, and 7 up-regulated DEGs involved in starch and sucrose metabolism, glycolysis/gluconeogenesis, TCA cycle, and oxidative phosphorylation were found, respectively, under DS. Then we compared DEGs related to energy metabolism in the aboveground part of bermudagrass under SS and DS for 7 d and found that 64 down-regulated DEGs and 12 up-regulated DEGs were co-expressed under both SS and DS (Table 2).

Similarly, we compared the DEGs in underground part of bermudagrass under SS and DS for 7 d. The amount of up-regulated DEGs (175 in total) related to energy metabolism was much larger than that of the down-regulated DEGs (1 in total) in underground tissues under SS for 7d (Figure 4Bb). Among these DEGs, we found that 25, 14, 45, and 91 up-regulated DEGs were involved in starch and sucrose metabolism, glycolysis/gluconeogenesis, TCA cycle, and oxidative phosphorylation, respectively, although 1 beta-fructofuranosidase (INV) in starch and sucrose metabolism was down-regulated. However, two up-regulated DEGs and one down-regulated DEG in the underground part of bermudagrass were related to starch and sucrose metabolism and glycolysis/gluconeogenesis, respectively, under DS (Figure 4Bd). This demonstrated that the expression of almost all the genes related to energy metabolism remains unchanged in the underground part of bermudagrass under DS for 7 d. We did not find co-expressed DEGs related to energy metabolism in underground tissues of bermudagrass under SS and DS for 7 d. Surprisingly, when we analyzed the DEGs in underground tissues of bermudagrass under SS, we obtained exactly the opposite result from the aboveground (Figure 4Ba,b), which suggested that the response of aboveground and underground tissues of bermudagrass to SS stress was completely different.

### 2.5. DEGs Related to Internal Gas Diffusion: Aerenchyma

**Figure 5:**
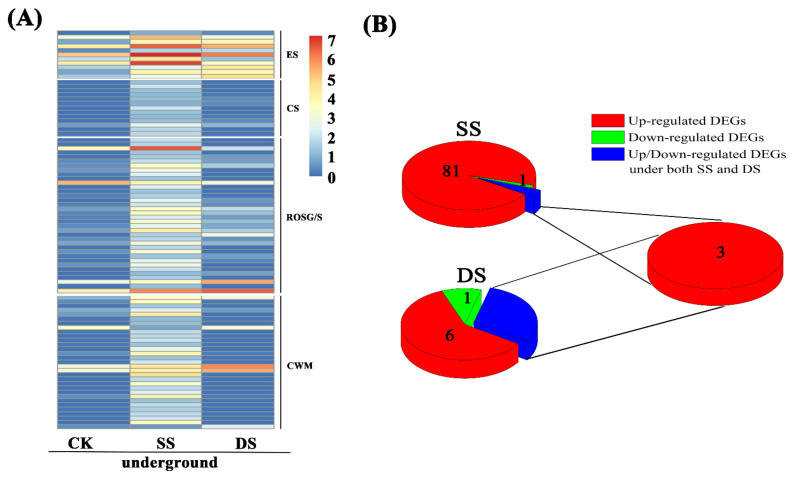
The expression of differentially expressed genes (DEGs) related to aerenchyma in underground tissues of bermudagrass. (**A**) Heatmap of DEGs expression profiles under control (CK), shallow submergence (SS), and deep submergence (DS). The *x* axis represents different treatments. The *y* axis represents the value of relative expression level (log_2_ (FPKM + 1)). (**B**) The number of DEGs expression under SS and DS. The red parts indicate up-regulated DEGs; the green parts indicate down-regulated DEGs; the blue parts indicate co-expression of DEGs under both SS and DS. FPKM: fragments per kilobase of transcript per million mapped reads; ES: ethylene signaling; CS: calcium signaling; ROSG/S: ROS generation/scavenging; CWM: cell wall modification.

We screened out some DEGs related to aerenchyma formation through annotated genes in the GO, KEGG, NR, Pfam, and Swiss-Prot databases, mainly including ethylene signaling, calcium signaling, ROS generation/scavenging, as well as cell wall modification. Further, the overall expression pattern of DEGs related to aerenchyma formation was analyzed by using the heatmap method (Figure 5A). We summarized that there were 84 and 9 up-regulated DEGs and 1 and 1 down-regulated DEGs in underground under SS and DS, respectively (Figure 5B). Among these DEGs, only three DEGs (one ethylene-responsive transcription factor (ERTF, encoded by *c149807.graph_c0*), one glutathione S-transferase (GST, encoded by *c105520.graph_c0*), and one myo-inositol oxygenase (MIOX, encoded by *c152237.graph_c1*)) were co-expressed in underground tissues of bermudagrass under both SS and DS, and all of them were up-regulated.

**Figure 6:**
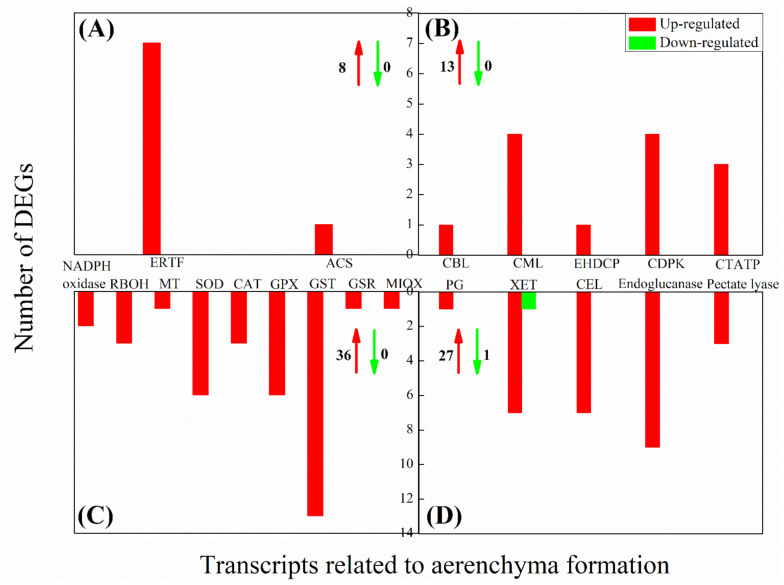
The expression of differentially expressed genes (DEGs) related to aerenchyma formation (ethylene signaling (**A**), calcium signaling (**B**), ROS generation/scavenging (**C**), and cell wall modification (**D**)) in underground tissues of bermudagrass under shallow submergence (SS). ERTF: ethylene-responsive transcription factor; ACS: 1-aminocyclopropane-1-carboxylic acid synthase; CBL: calcineurin B-like protein; CML: calcium-binding proteins and calmodulin-like protein; EHDCP: EH domain-containing protein; CDPK: calcium/Calmodulin-dependent protein kinases; CTATP: ca^2+^-transporting ATPase; RBOH: respiratory burst oxidase homolog; MT: metallothionein; SOD: superoxide dismutase; CAT: catalase; GPX: glutathione peroxidase; GST: glutathione S-transferase; GSR: glutathione reductase; MIOX: myo-inositol oxygenase; PG: polygalacturonase; XET: xyloglucan endotransglucosylase; CEL: cellulase.

**Figure 7:**
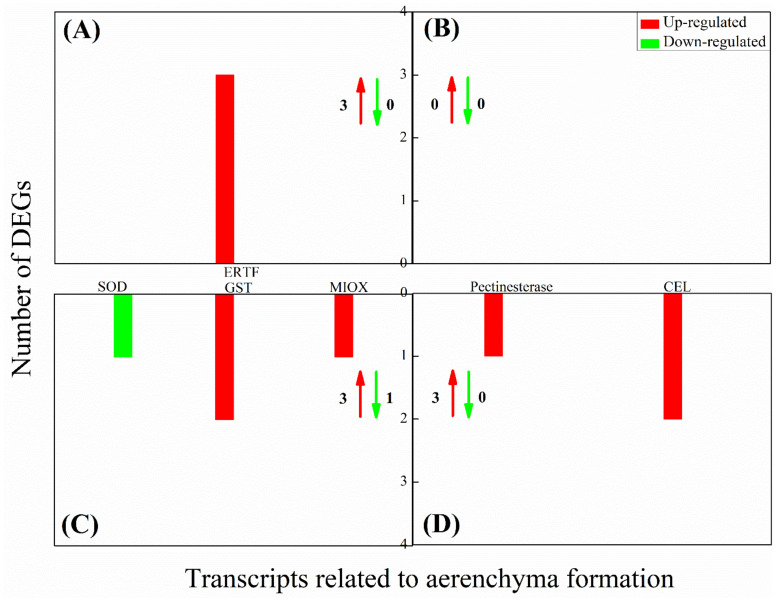
The expression of differentially expressed genes (DEGs) related to aerenchyma formation (ethylene signaling (**A**), calcium signaling (**B**), ROS generation/scavenging (**C**), and cell wall modification (**D**)) in underground tissues of bermudagrass under deep submergence (DS). ERTF: ethylene-responsive transcription factor; SOD: superoxide dismutase; GST: glutathione S-transferase; MIOX: myo-inositol oxygenase; CEL: cellulase.

The expression level of 7 and 3 ERTF genes was markedly up-regulated in underground tissues of bermudagrass under SS and DS, respectively (Figure 6A and Figure 7A). The expression intensities (log_2_ FC) of those up-regulated DEGs were all greater than 2.31, and the maximum reached 4.44. At the same time, one 1-aminocyclopropane-1-carboxylic acid (ACC) synthase (ACS) was up-regulated in underground tissues under SS (Figure 6A). Respectively, 1, 4, 1, 4, and 3 DEGs (13 in total) involved in calcineurin B-like protein (CBL), calcium-binding proteins and calmodulin-like protein (CML), EH domain-containing protein (EHDCP), calcium/calmodulin-dependent protein kinases (CDPK) and ca^2+^-transporting ATPase (CTATP) were up-regulated in underground tissues under SS (Figure 6B). However, the DEGs related to calcium signaling were not detected in underground tissues of bermudagrass under DS. Meanwhile, 36 (36 in total) and 3 (out of 4 in total) up-regulated expressed DEGs related to ROS generation/scavenging in underground tissues of bermudagrass under SS and DS were finally identified, respectively (Figure 6C and Figure 7C). In total, 2, 3, 1, 6, 3, 6, 13, 1, and 1 up-regulated DEGs involved in nicotinamide adenine dinucleotide phosphate (NADPH) oxidase, NADPH oxidase respiratory burst oxidase homolog (RBOH), metallothionein (MT), superoxide dismutase (SOD), catalase (CAT), glutathione peroxidase (GPX), GST, glutathione reductase (GSR), and MIOX under SS, respectively (Figure 6C). Additionally, there were one down-regulated and two and one up-regulated DEGs involved in SOD, GST, and MIOX under DS, respectively (Figure 7C). These indicated that ROS contents were decreased in underground tissues of bermudagrass under DS. Furtherore, 28 and 3 DEGs related to cell wall degradation of hydrolases were observed under SS and DS, respectively (Figure 6D and Figure 7D). These up-regulated DEGs were primarily related to encoded polygalacturonase (PG), xyloglucan endotransglucosylase (XET), cellulase (CEL), endoglucanase, pectate lyase, and pectinesterase.

## 3. Discussion

### 3.1. Modulation of Photosynthetic Pathways under SS and DS

Photosynthesis is an important basic physiological process for the growth and biomass accumulation of higher plants. It is also one of the most sensitive physiological processes of plants in response to flooding stress. Photosynthesis mainly includes the light reaction stage and carbon-fixation reaction stage, involving light absorption, electron transport, photophosphorylation, and carbon assimilation.

Our study identified that the genes involved in photosynthesis light system apparatus were preferentially down-regulated under both SS and DS conditions (Figure 3). Chlorophyll is an important substance for light energy capture, transmission, and transformation, and its content can directly reflect the photosynthetic capacity of plants [38]. Genes for all 15 reactions in the chlorophyll biosynthesis from glutamyl-tRNA to chlorophylls a and b were identified. The enzyme activity of seven reactions was markedly down-regulated in the present study, including hydroxymethylbilane synthase (encoded by *HMBS*), uroporphyrinogen decarboxylase (encoded by *UROD*), protoporphyrinogen/coproporphyrinogen III oxidase (encoded by *PPOX*), magnesium chelatase subunit I (encoded by *chlI*), magnesium-protoporphyrin IX monomethylester cyclase (encoded by *chlE*), protochlorophyllide reductase (encoded by *por*), and chlorophyllide a oxygenase (encoded by *CAO*) [39]. Meanwhile, the geranylgeranyl reductase encoded by *chlP* was involved in phytyl moiety for chlorophyll synthesis [40,41]. It is widely accepted that light can also be captured by the largest subunits CP43 (encoded by *PsbC*) of PSII, in addition to light-harvesting chlorophyll protein complex (LHC), and can then be transferred to a reaction center complex containing chlorophyll [42,43]. Our present results show that nine genes encoding LHC and one gene encoding CP43 were significantly down-regulated under flooding conditions (Figure 3), implying that the light-capturing was greatly inhibited. It is generally known that light reactions include four main protein complexes, such as PSII, cytochrome b6/f complex, PS I, and F-type ATPase. In our present study, we found that there are 20 down-regulated genes encoding these protein complexes. Ye et al. (2015) [32] showed that three proteins identified as Cyt b6f and one ATP synthase protein were inhibited under submergence in bermudagrass. To sum up, the light reaction stage was severely affected by flooding stress.

Dark reactions can convert active chemical energy into stable chemical energy and can store it in different forms of sugar. In a flooding environment, reduced intercellular carbon dioxide (CO_2_) concentration caused by stomata closure and low gas exchange severely affect carbon fixation [44]. In our present study, we observed that the enzyme activity of phosphoenolpyruvate carboxylase (encoded by *ppc*) and malate dehydrogenase (encoded by *NADP^+^*, *MDH*) were markedly down-regulated during the C4 dicarboxylic acid cycle (Figure 3); moreover, deactivation was displayed in phosphorribulokinase and other enzymes such as phosphoglycerate kinase, glyceraldehyde-3-phosphate dehydrogenase (NADP^+^), fructose-1,6-bisphosphatase, sedoheptulose-bisphosphatase, and so on during the Calvin cycle in this study (Figure 3). Locke et al. (2018) also demonstrated that key genes related to photosynthesis (i.e., photosystem subunits, rate-limiting enzymes of the Calvin cycle) were down-regulated during submergence in rice (*Oryza sativa* L.) [45]. This is because the bermudagrass roots’ ability to absorb water and mineral elements is significantly affected by submergence stress, which results in the blockage of the nutrient elements required by the synthetic process of protein chlorophyll in the leaves and thus leads to the significantly reduced activities of photosynthesis-related enzymes. This ultimately causes a decline of photosynthesis in bermudagrass [16]. From the above, the expression level of DEGs related to photosynthesis (light reaction and dark reaction) were inhibited, hence leading to a reduction in the photosynthetic capacity and photosynthetic products of bermudagrass. Thus, this implies that the nutrient reserves were reduced in bermudagrass, threatening its survival.

### 3.2. Modulation of Energy Metabolism Pathways under SS and DS

**Figure 8:**
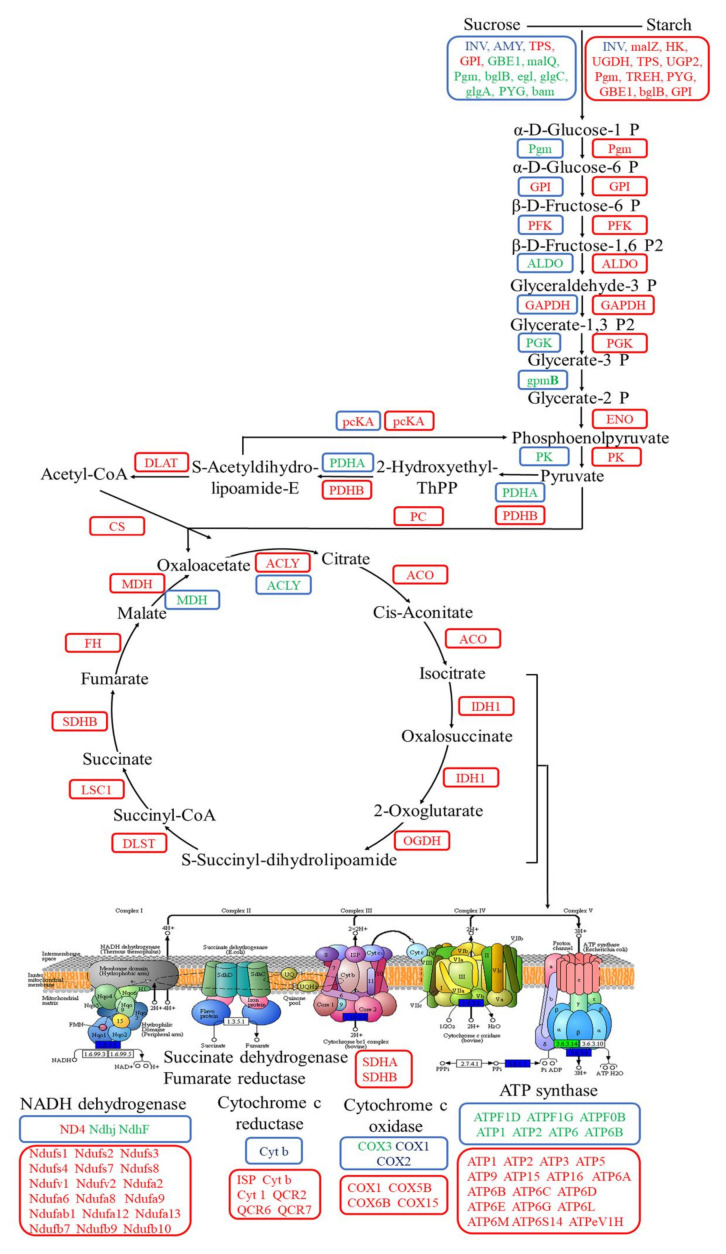
The expression of differentially expressed genes (DEGs) related to energy metabolism in aboveground and underground tissues of bermudagrass under shallow submergence (SS). The blue box represents the aboveground part, and the red box represents the underground part. Red letters represent up-regulated expression; green letters represent down-regulated expression; blue letters represent both up-regulated and down-regulated expression. INV: beta-fructofuranosidase; bglB: beta-glucosidase; AMY: α-amylase; egl: endoglucanase TPS: trehalose 6-phosphate synthase/phosphatase; glgC: glucose-1-phosphate adenylyl transferase; GPI: glucose-6-phosphate isomerase; glgA: starch synthase; GBE1: 1,4-alpha-glucan branching enzyme; PYG: glycogen phosphorylase; malQ: 4-alpha-glucanotransferase; bam: beta-amylase; Pgm: phosphoglucomutase; malZ: alpha-glucosidase; HK: hexokinase; TREH: alpha, alpha-trehalase; UGDH: UDP glucose 6-dehydrogenase; GBE1: 1,4-alpha-glucan branching enzyme; UGP2: UTP-glucose-1-phosphate uridylyl transferase; PFK: phosphoglycerate kinase; ALDO: fructose-bisphosphate aldolase, class I; GAPDH: glyceraldehyde 3-phosphate dehydrogenase; PGK: phosphoglycerate kinase; gpmB: probable phosphoglycerate mutase; ENO: enolase; pcKA: phosphoenolpyruvate carboxykinase (ATP); PK: pyruvate kinase; PDHA: pyruvate dehydrogenase E1 component alpha subunit; PDHB: pyruvate dehydrogenase E1 component beta subunit; PC: pyruvate carboxylase; DLAT: pyruvate dehydrogenase E2 component (dihydrolipoamide acetyltransferase); CS: citrate synthase; ACLY: ATP citrate (pro-S)-lyase; ACO: aconitate hydratase; IDH1: isocitrate dehydrogenase; OGDH: 2-oxoglutarate dehydrogenase E1 component; DLST: 2-oxoglutarate dehydrogenase E2 component (dihydrolipoamide succinyltransferase); LSC1: succinyl-CoA synthetase alpha subunit; SDHB: succinate dehydrogenase (ubiquinone) iron-sulfur subunit; FH: fumarate hydratase, class II; MDH: malate dehydrogenase; ND4: NADH-ubiquinone oxidoreductase chain 4; NdhJ: NAD(P)H-quinone oxidoreductase subunit J; NdhF: NAD(P)H-quinone oxidoreductase subunit 5; Cyt b: ubiquinol-cytochrome c reductase b subunit; COX1–3: cytochrome c oxidase subunit 1–3; Ndufs1–4, 7, 8: NADH dehydrogenase (ubiquinone) Fe-S protein 1–4, 7, 8; Ndufv 1–2: NADH dehydrogenase (ubiquinone) flavoprotein 1–2; Ndufa 2, 6, 8, 9: NADH dehydrogenase (ubiquinone) 1 alpha subcomplex subunit 2, 6, 8, 9; Ndufab 1: NADH dehydrogenase (ubiquinone) 1 alpha/beta subcomplex 1, acy-carrier protein; Ndufa12–13: NADH dehydrogenase (ubiquinone) 1 alpha subcomplex subunit 12–13; Ndufb 7, 9, 10: NADH dehydrogenase (ubiquinone) 1 beta subcomplex subunit 7, 9, 10; SDHA: succinate dehydrogenase (ubiquinone) flavoprotein subunit; SDHB: succinate dehydrogenase (ubiquinone) iron-sulfur subunit; ISP: ubiquinol-cytochrome c reductase iron-sulfur subunit; Cyt 1: ubiquinol-cytochrome c reductase c1 subunit; QCR 2: ubiquinol-cytochrome c reductase core subunit 2; QCR 6, 7: ubiquinol-cytochrome c reductase subunit 6, 7; COX5B, 6B: cytochrome c oxidase subunit 5b, 6b; COX15: heme a synthase; ATPF0B, 1D, 1G, 6, 6B: F-type H^+^-transporting ATPase subunit b, delta, gamma, a, B; ATP1–3, 5, 9, 15, 16: F-type H^+^-transporting ATPase subunit alpha, beta, gamma, o, c, epsilon, delta; ATP6A-E, G, M, S14: V-type H^+^-transporting ATPase subunit A-C, d, E, G, D, F; ATP6L: V-type H^+^-transporting ATPase 16kDa proteolipid subunit; ATPeV1H: V-type H^+^-transporting ATPase subunit H.

Carbohydrates are the main products of plant photosynthesis and can be divided into structural carbohydrates and non-structural carbohydrates. In general, structural carbohydrates (lignin, cellulose, etc.) play an important role in constructing plant morphology. At the same time, non-structural carbohydrates (soluble sugars and starches) are the main energy source of plants, which is of great significance for plants’ survival under flooding stress [46,47,48,49]. Generally, plants can change the cellulate osmotic potential to tolerate adverse stress by adjusting soluble sugar content [50]. The results of our study revealed that most hydrolase activities related to starch and sucrose metabolism were significantly down-regulated in aboveground tissues of bermudagrass under SS, including endoglucanase (encoded by *egl*) and beta-glucosidase (encoded by *bglB*) that hydrolyze cellulose, glucose-1-phosphate adenylyltransferase (encoded by *glgC*), 4-alpha-glucanotransferase (encoded by *malQ*), beta-amylase (encoded by *bam*), and phosphoglucomutase (encoded by *pgm*). It is worth noting that most of the underground hydrolase activities were significantly up-regulated in SS (Figure 8). This illustrates the fact that the soluble sugar content was markedly reduced in aboveground and increased in underground tissues of bermudagrass under SS compared with that under CK (Figure 8). These results indicate that the hydrolases play an important role in the adaption of bermudagrass to SS stress.

Additionally, soluble sugars are used as a direct energy source and can produce large amounts of ATP through the Embdem–Meyerhof–Parnas (EMP)-TCA cycle under aerobic conditions. However, EMP may become the primary energy source when the aerobic respiration pathway is inhibited under flooding conditions (hypoxia and/or anaerobic) [51]. Breaking down glucose into pyruvate and generating ATP is known as EMP and involves 10 consecutive enzymatic reactions. The following 10 enzymes are involved in these enzymatic reactions: hexokinase (HXK), glucose-6-phosphate isomerase (GPI), phosphofructokinase (PFK), D-fructose-1,6-diphosphate (FBA), triose-phosphate isomerase (TPI), glyceraldehyde-3-phosphate dehydrogenase (GAPDH), phosphoglycerate kinase (PGK), phosphoglyceratemutase (PGAM), enolase (ENO), and pyruvate kinase (PK) [52]. In the presence of O_2_, pyruvate will undergo complete oxygenolysis through TCA and oxidative phosphorylation and will finally produce CO_2_, H_2_O, and ATP [53]. In this study, we ascertained that the expression of several key enzymes involved in EMP were significantly down-regulated in aboveground tissues of bermudagrass under SS, such as FBA (encoded by *ALDO*), PGK (encoded by *PGK*), PGAM (encoded by *gpmB*), and PK (encoded by *PK*). However, GPI (encoded by *GPI*), PFK (encoded by *PFK*), and GAPDH (encoded by *GAPDH*) enzyme activities were up-regulated. Afterward, the activities of pyruvate dehydrogenase (encoded by *PDHA*) in the gluconeogenesis and malate dehydrogenase (encoded by *MDH*) in the TCA cycle were down-regulated (Figure 8). Pyruvate can be catalyzed by pyruvate dehydrogenase to generate acetyl-coA, and its condensation with oxaloacetate is considered as the first step to initiate the TCA cycle. Meanwhile, malate dehydrogenase catalyzes the conversion of malate to oxaloacetate and plays a key role in the new turn of the TCA cycle [54]. Pyruvate dehydrogenase and malate dehydrogenase were down-regulated in our present study, implying that the TCA cycle was blocked. Oxidative phosphorylation is an important stage in the TCA cycle, where a large number of genes (such as *ATPF1D*, *ATPF1G*, *ATPF0B*, *ATP1*, *ATP2*, *ATP6*, and *ATP6B*) related to ATP synthase were down-regulated as well (Figure 8). Thus, ATP synthesis and energy metabolism were apparently weakened. As a whole, the aboveground tissues of bermudagrass responded to SS stress by reducing the content of soluble sugar and cell respiration.

**Figure 9:**
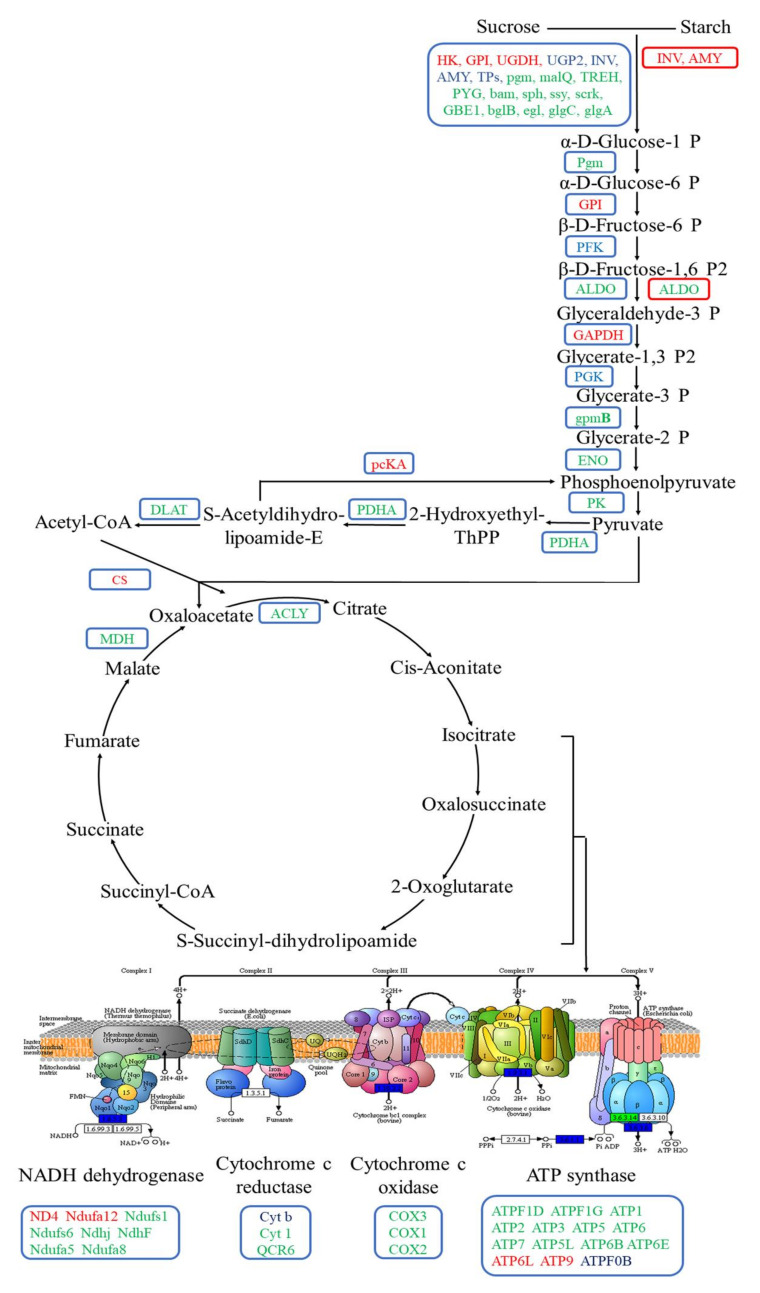
The expression of differentially expressed genes (DEGs) related to energy metabolism in aboveground and underground tissues of bermudagrass under deep submergence (DS). The blue box represents the aboveground, and the red box represents the underground. Red letters represent up-regulated expression; green letters represent down-regulated expression; blue letters represent both up-regulated and down-regulated expression. HK: hexokinase; GPI: glucose-6-phosphate isomerase; UGDH: UDP glucose 6-dehydrogenase; INV: beta-fructofuranosidase; AMY: α-amylase; TPs: trehalose 6-phosphate synthase/phosphatase; UGP2: UTP-glucose-1-phosphate uridylyl transferase; sph: sucrose-phosphate; ssy: sucrose synthase; scrk: fructokinase; GBE1: 1,4-alpha-glucan branching enzyme; malQ: 4-alpha-glucanotransferase; TREH: alpha-trehalase; pgm: phosphoglucomutase; bglB: beta-glucosidase; egl: endoglucanase; glgC: glucose-1-phosphate adenylyl transferase; glgA: starch synthase; PYG: glycogen phosphorylase; bam: beta-amylase; PFK: phosphoglycerate kinase; ALDO: fructose-bisphosphate aldolase, class I; GAPDH: glyceraldehyde 3-phosphate dehydrogenase; PGK: phosphoglycerate kinase; gpmB: probable phosphoglycerate mutase; ENO: enolase; pcKA: phosphoenolpyruvate carboxykinase (ATP); PK: pyruvate kinase; PDHA: pyruvate dehydrogenase E1 component alpha subunit; DLAT: pyruvate dehydrogenase E2 component (dihydrolipoamide acetyltransferase); CS: citrate synthase; ACLY: ATP citrate (pro-S)-lyase; MDH: malate dehydrogenase; ND4: NADH-ubiquinone oxidoreductase chain 4; Ndufa12: NADH dehydrogenase (ubiquinone) 1 alpha subcomplex subunit 12; Ndufs1, 6: NADH dehydrogenase (ubiquinone) Fe-S protein 1, 6; NdhJ: NAD(P)H-quinone oxidoreductase subunit J; NdhF: NAD(P)H-quinone oxidoreductase subunit 5; Ndufa5, 8: NADH dehydrogenase (ubiquinone) 1 alpha subcomplex subunit 5, 8; Cyt b: ubiquinol-cytochrome c reductase b subunit; Cyt 1: ubiquinol-cytochrome c reductase c1 subunit; QCR 6: ubiquinol-cytochrome c reductase subunit 6; COX1–3: cytochrome c oxidase subunit 1–3; ATPF0B, 1D, 1G: F-type H^+^-transporting ATPase subunit b, delta, gamma; ATP1–3, 5–7, 9, 5L: F-type H^+^-transporting ATPase subunit alpha, beta, gamma, o, a, d, c, g; ATP6B, E: V-type H^+^-transporting ATPase subunit B, E; ATP6L: V-type H^+^-transporting ATPase 16kDa proteolipid subunit.

As shown in transcriptome analysis, the energy metabolism pathway was differentially regulated between aboveground and underground tissues of bermudagrass in SS (Figure 9). The expression of a large number of genes related to the energy metabolism pathway in the underground tissues was significantly up-regulated. For instance, the expression of genes related to starch and sucrose were up-regulated, including *malZ*, *TREH*, *UGDH*, *bglB*, and so on. These would lead to the accumulation of reaction substrates in the glycolysis pathway and then would improve the reaction rate. Meanwhile, the expression of eight key genes (including *HK*, *GPI*, *PFK*, *ALDO*, *GAPDH*, *PGK*, *ENO*, and *PK*) related to glycolysis were significantly up-regulated. In addition, the expression of genes related to pyruvate dehydrogenase (encoded by *PDHA*, *DLAT*) and pyruvate carboxylase (encoded by *PC*) was significantly up-regulated (Figure 9). Therefore, the amount of acetyl-coA and oxaloacetate that was catalyzed by pyruvate would be accumulated. Additionally, notorious underground effects of hypoxia are associated with flooding stress, in which the transformation of plant respiratory metabolism changes from aerobic respiration to anaerobic respiration. Interestingly, the gene expression in TCA cycle and oxidative phosphorylation were significantly up-regulated in underground tissues of bermudagrass under SS in our present study. The genes such as *CS*, *ACO*, *IDH1*, *OGDH*, *DLST*, *LSC1*, *SDHB*, *FH*, *MDH*, and *ACLY* that encode nine enzymes involved in the TCA cycle were up-regulated in this present study, including citrate synthase (CS, encoded by *CS*), isocitrate dehydrogenase (IDH, encoded by *IDH1*), and α-ketoglutarate dehydrogenase complex (α-KGDH, encoded by *OGDH*, *DLST*) that determine the speed and direction of the TCA. Nonetheless, genes encoding NADH dehydrogenase (e.g., *Ndufs1–4*, *Ndufv1–2*), succinate dehydrogenase fumarate reductase (*SDHA*, *SDHB*), cytochrome c reductase (e.g., *ISP*, *Cyt b*), cytochrome c oxidase (*COX1*, *COX5B*), and ATP synthase (e.g., *ATP1–3*) were related to oxidative phosphorylation (Figure 9). This is most likely because the root of bermudagrass is directly affected by submergence stress, and it can obtain more energy to maintain normal life activities by enhancing cellular respiration. At the same time, carbon transfer occurs from the aboveground to the underground in bermudagrass to ensure adequate substrate supply. There is no doubt that there was relatively sufficient oxygen in the roots of bermudagrass under SS. Therefore, these genes that encode aerenchyma play a key role in roots.

Under DS, we discovered that the genes encoding most hydrolase activities and starch-sucrose synthase were significantly down-regulated in the aboveground part of bermudagrass (Figure 9). This implies that the content of soluble sugar and starch in the aboveground part of bermudagrass was remarkably reduced under DS. The change of soluble sugar content and starch content in our current study was consistent with previous studies [17]. As opposed to the aboveground, the expression of genes regulating INV and α-amylase (AMY) were significantly up-regulated in the underground. Sucrose and starch can be hydrolyzed to fructose and glucose by INV and AMY, respectively. The increase of glucose and fructose provided the energy for EMP and other processes in the underground part of bermudagrass [55]. It is worth noting that there was no significant change in gene expression during glycolysis, TCA cycle, and oxidative phosphorylation under DS after 7 d. The former study documented that ethanol dehydrogenase activity in bermudagrass roots increased under submergence and increased submergence depth [22]. However, the differential expression of genes related to anaerobic respiration enzyme activity (such as pyruvate decarboxylase, ethanol dehydrogenase, and lactate dehydrogenase) was not found in the present study reflecting that the aerobic respiration was not seriously affected after 7 d under DS in our present research. In this context, we believe that developed aerenchyma in roots plays an extremely important role in DS.

### 3.3. Modulation of Aerenchyma Pathways under SS and DS

From the energy metabolism pathway, there was sufficient oxygen for aerobic respiration in bermudagrass roots under both SS and DS after 7 d treatment, which is attributed to the formation of aerenchyma that can effectively improve a plant’s aeration conditions and facilitate its tolerance to flooding [29,56].

**Figure 10:**
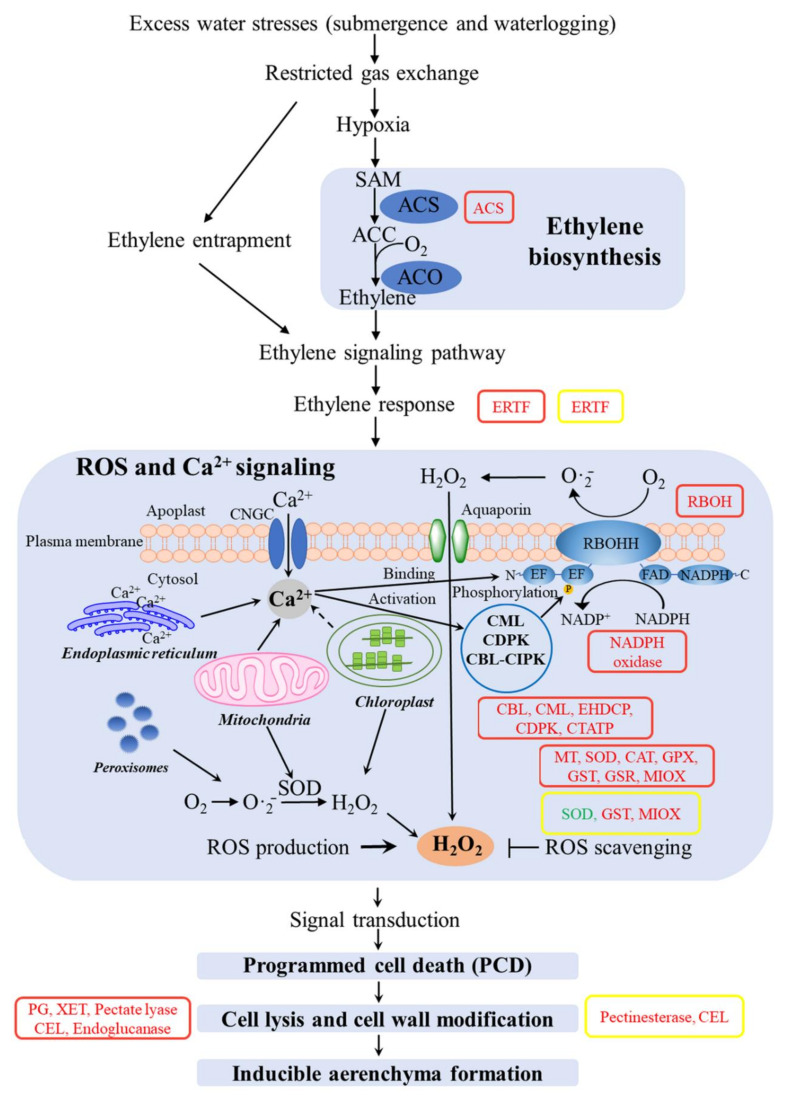
The expression of differentially expressed genes (DEGs) related to aerenchyma formation in underground tissues of bermudagrass under shallow submergence (SS) (Red box) and deep submergence (DS) (Yellow box). Red letters represent up-regulated expression; green letters represent a down-regulated expression. ACS: 1-aminocyclopropane-1-carboxylic acid synthase; ERTF: ethylene-responsive transcription factor; RBOH: respiratory burst oxidase homolog; CBL: calcineurin B-like protein; CML: calcium-binding proteins and calmodulin-like protein; EHDCP: EH domain-containing protein; CDPK: calcium/calmodulin-dependent protein kinases; CTATP: ca^2+^-transporting ATPase; MT: metallothionein; SOD: superoxide dismutase; CAT: catalase; GPX: glutathione peroxidase; GST: glutathione S-transferase; GSR: glutathione reductase; MIOX: myo-inositol oxygenase; PG: polygalacturonase; XET: xyloglucan endotransglucosylase; CEL: cellulase.

In order to understand the genes that regulated the root aerenchyma formation in bermudagrass under SS and DS, a representative figure was made by combining five stages of cell death in the root cortex and the expression of DEGs (Figure 10). We analyzed and summarized the DEGs of the root aerenchyma formation process under SS. First, the genes that regulate rate-limiting enzyme ACS were significantly up-regulated in ethylene biosynthesis, which promoted ACC synthesis of ethylene biosynthesis direct precursor and finally resulted in the increase of ethylene synthesis (Figure 10). The results of the present study were similar to the previous studies that found that ACS expression level was strongly increased in rice roots under stagnant conditions [57]. Additionally, ethylene entrapment further increased the accumulation of ethylene in roots. In addition, a large amount of genes related to ERTF were up-regulated in our study. Some ERTFs play a key role in a plant’s response to abiotic stress and the interaction of signal pathways [58]. Finally, PCD was stimulated during lysigenous aerenchyma formation [59].

There is compelling evidence that Ca^2+^ and ROS acted as essential second messengers regulating signal transduction pathways in plants. The initial Ca^2+^ influx mediated by plasma membrane ion channels is considered to be the key to adaptive signaling and a prerequisite for ROS production [60,61]. Ca^2+^-binding to the EF-hand motifs and Ca^2+^-dependent protein kinases (CBL-CBL interacting protein kinases (CIPK), CDPK) phosphorylation synergistically activates the expression of the RBOH gene in the Ca^2+^-ROS signaling network [62,63,64]. Accumulating evidence suggests that RBOH is a major source of ROS in plants, was responsible for the conversed O_2_ to the superoxide anion radical (O_2_$\bar{\cdot }$), and sequentially hydrogen peroxide (H_2_O_2_) [59,65]. In plant cells, variation of Ca^2+^ and generated H_2_O_2_ would induce the occurrence of PCD procession and eventually induce aerenchyma formation [66,67,68]. Previous studies have shown that genes related to Ca^2+^ signaling (i.e., CBL, CML and CDPK) and ROS signaling (i.e., RBOH, MT) were significantly up-regulated during the formation of lysigenous aerenchyma in roots of maize (*Zea mays* ssp. *mays*) [30] and wheat (*Triticum aestivum*) [68]. Similarly, our present results reveal that Ca^2+^-dependent protein kinases (CBL-CIPK, CDPK) and RBOH genes were also significantly up-regulated, which leads to an increase in H_2_O_2_ levels in the apoplast and cytosol (Figure 10). Additionally, ROS plays a dual role in plants as both signaling molecules in aerenchyma formation and as a toxic effect to plant cells. The tightened regulation of ROS steady-state level by the “ROS gene network” allows this duality in function to exist in plants [69,70]. In recent years, the importance of ROS-scavenging enzymes such as SOD, CAT, GPX, GST, and GSR to detoxified O_2_$\bar{\cdot }$ and H_2_O_2_ have gained extensive support [69,71,72]. Additionally, MT was a key factor in ROS scavengers [73]. However, the genes associated with ROS-scavenging enzymes (such as SOD, CAT, GPX, GST, and GSR) and ROS scavenger factor (i.e., MT) were significantly up-regulated, effectively removing excessive accumulated ROS and mitigating the damage of ROS to the cell biomembrane system, thereby improving the submergence tolerance of bermudagrass (Figure 10).

Cell wall degradation promotes cortical cells to connect radially with each other to form aerenchyma. Rajhi et al. (2011) [30] reported that waterlogging induced the expression of genes related to cell wall modification (such as XET, CEL, PG, and pectate lyase) in maize roots. Kong et al. (2008) [74] documented that the expression of the XET gene was synchronized with aerenchyma formation to a certain extent. CEL and pectinase activities increase sharply under hypoxia stress, degrading cell walls and promoting aerenchyma formation [75]. Similarly, we also found that several genes related to cell wall loosening and degradation (e.g., XET, CEL, PG, pectate lyase, and endoglucanase) were up-regulated and then promoted cell wall disintegration (Figure 10), which was consistent with previous results. Taken as a whole, the SS treatment group could limit oxygen availability and ethylene accumulation. Later, extracellular Ca^2+^ influx stimulated a signaling cascade, including the activation of NADPH oxidases. Then, the activity of cell wall degrading enzymes was increased and ultimately induced aerenchyma formation in bermudagrass.

Previous studies showed that aerenchyma formation in *Juncus effusus* roots [27] does not need ethylene to trigger the PCD processes. Recently, Khan et al. (2020) [76] found that increased intensity of submergence induces ROS overproduction and stimulates the activity of genes associated with ethylene production and cell wall degrading enzymes, resulting in aerenchyma formation. Furthermore, ethylene was known to play an important role in triggering cell wall degrading enzymes (particularly cellulase) during aerenchyma formation. The DEGs during aerenchyma formation of bermudagrass in DS were also analyzed (Figure 10). Surprisingly, as opposed to aerenchyma formation in SS (ethylene-induced RBOH-mediated ROS production), the Ca^2+^-ROS signaling network-related genes did not change in DS. Only some genes related to both ERTF and cell wall modification were up-regulated. Apparently, the aerenchyma formed did not require ethylene to trigger the cortical cells’ PCD process in the roots of bermudagrass under DS.

## 4. **Materials and Methods**

### 4.1. Plant Materials and Experimental Treatments

Bermudagrass and purple soil were collected from the hydro-fluctuation belt of Ruxi river from Shibao town, in the Three Gorges Reservoir, China (107°32′–108°14′ E, 30°03′–30°35′ N) [77,78,79]. This experiment adopted the potted control test. The size of the pot was 8 cm high and 8.8 cm in diameter. The cutting cultivation of bermudagrass began on 12 April 2018, and it was cut into 10 cm long segments for planting in the experimental garden. After that, the cuttings were placed under the experimental booth of the State Key Laboratory of Eco-environments in the Three Gorges Reservoir Region for 60 d acclimation growth under the same conditions.

Two-month-old cuttings were used for the three different water gradients. The treatments included: (i) CK: normal water supply, with soil moisture content being 60–63% of soil field water capacity; (ii) SS: submergence 10 cm above the soil surface; (iii) DS: submergence over the top of the plant 100 cm. The aboveground (all leaves and stems above the surface of the soil) and underground (all adventitious roots below the surface of the soil) of bermudagrass under each treatment were separately collected after 7 d treatment. The collected samples were then immediately frozen in liquid nitrogen and taken back to the laboratory for storage at −80 °C until analysis.

### 4.2. RNA Isolation and Sequencing/RNA-seq Library Preparation and Sequencing

To acquire a global overview of tra(nscriptome in aboveground and underground tissues of bermudagrass, an equivalent amount of total RNA isolated from three aboveground tissues and three underground tissues were pooled, respectively [37]. Total RNA was isolated, following Parkhomchuk et al. (2009) [80], and extracted using the TRIzol^®^ Reagent (Invitrogen, Carlsbad, CA, USA). RNA purity and RNA concentration were checked by using the NanoPhotometer^®^ spectrophotometer (IMPLEN, Westlake Village, CA, USA) and Qubit^®^2.0 Fluorometer (Life Technologies, Carlsbad, CA, USA), respectively. The mRNA was purified from total RNA with poly-T oligo-attached magnetic beads. The library fragments were purified with AMPure XP system (Beckman Coulter, Beverly, MA, USA). Library quality was assessed on the Agilent Bioanalyzer 2100 system, and the cDNA library was sequenced in the Illumina Hiseq 2000 platform (BMKcloud, Beijing, China).

### 4.3. Identification and Functional Annotation of DEGs

We removed reads containing adapter, reads containing ploy-N, and low quality reads from raw data, then obtained the clean data (clean reads), and then calculated clean data (Q20, Q30, GC-content, and sequence duplication level). All the downstream analyses were based on clean data with high quality. Gene function was annotated during August 2018 based on the following databases: NR (NCBI non-redundant protein sequences), Pfam (Protein family), KOG/COG/eggNOG (Clusters of Orthologous Groups of proteins), Swiss-Prot (a manually annotated and reviewed protein sequence database), KEGG (Kyoto Encyclopedia of Genes and Genomes), and GO (Gene Ontology).

The edgeR program package adjusted the read counts in each sequenced library through one scaling normalized factor before differential gene expression analysis. The DEGseq (2010) R package was used to analyze the differential expression analysis of samples and used q-value to the adjusted *p*-value. The threshold for significantly differential expression was *q*-value < 0.005 and |log_2_ FC| > 1.

### 4.4. The KEGG Pathway Enrichment Analysis

KEGG database was used to understand high-level functions and utilities of the biological system (such as cell, organism, and ecosystem) from molecular-level information [81]. We tested the statistical enrichment of DEGs in KEGG pathways using KOBAS 2.0 [82].

## 5. Conclusions

Submergence inhibited the expression of genes related to the light and dark reaction stages of photosynthesis in aboveground tissues of bermudagrass, resulting in a decrease in photosynthetic products. Moreover, the expression of genes related to sucrose and starch hydrolase was also down-regulated, which ultimately led to a decrease in the soluble sugar content of the aboveground part of bermudagrass. In addition, genes encoding the glycolytic key enzyme (*Pgm*, *ALDO*, *gpmB*, and *PK*), TCA cycle (*PDHA*, *MDH*, and *ACLY*), and ATP synthase (ATPF1D, ATPF1G, ATP1–2, ATP 6, and ATP 6B) were down-regulated, suggesting that the respiration of aboveground tissues of bermudagrass was affected to a certain extent under submergence. However, the results show that a large number of genes involved in the sucrose and starch hydrolysis, glycolysis, TCA cycle, and oxidative phosphorylation of the underground part were up-regulated under SS, actively maintaining the normal physiological activities of bermudagrass. Moreover, the genes encoding INV and AMY were up-regulated. The DEGs related to the TCA cycle and oxidative phosphorylation were not detected in the underground part of bermudagrass under DS, suggesting that the developed aerenchyma in the roots plays an important role. As expected, the genes related to aerenchyma formation in bermudagrass roots were significantly expressed in the present study. The genes encoding ethylene signal, calcium signal transduction, ROS production/scavenging, and cell wall degradation were up-regulated in SS. Only some genes encoding ethylene response, ROS scavenging, and cell wall degradation were up-regulated in DS. These results suggest that the underlying molecular mechanisms of aerenchyma formation induced by SS and DS were different. This study thoroughly explored the regulatory genes underlying the physiological responses of the submergence-tolerant bermudagrass plant. More importantly, some genes related to submergence tolerance were identified, providing a theoretical basis for subsequent genetic improvement of the submergence tolerance of bermudagrass.

## Figures and Tables

**Table 1:** Summary of sequencing quality evaluation and assembly statistics in aboveground and underground tissues of bermudagrass under different water regimes for 7 d.

Sample	Base Number	GC Content	% ≥ Q30	Clean Reads	Mapped Reads	Mapped Ratio
CK aboveground	7,708,624,970	53.06%	88.32%	25,745,275	19,292,852	74.94%
CK underground	8,084,087,134	52.92%	88.99%	26,992,331	20,142,196	74.62%
SS aboveground	6,837,998,294	53.65%	89.31%	22,842,105	16,743,522	73.30%
SS underground	7,941,119,312	53.13%	88.97%	26,520,179	19,451,568	73.35%
DS aboveground	6,968,619,394	55.04%	89.19%	23,287,321	16,828,567	72.26%
DS underground	6,834,848,554	52.71%	89.00%	22,825,956	16,885,731	73.98%

Note: CK: control; SS: shallow submergence; DS: deep submergence; Base Number: clean data total base number; GC Content: clean data GC content; % ≥ Q30: the percentage of bases with clean data mass value of 30 or greater; Clean Reads: clean reads number, pair-end; Mapped Reads: mapped reads number, pair-end; Mapped Ratio: the proportion of mapped reads to clean reads.

**Table 2:** The co-expressed differentially expressed genes (DEGs) related to energy metabolism in aboveground tissues of bermudagrass under shallow submergence (SS) and deep submergence (DS) for 7 d.

Name	Definition	Expression	Number
*bglB*	beta-glucosidase	down-regulated	14
*egl*	endoglucanase	down-regulated	2
*glgA*	starch synthase	down-regulated	3
*glgC*	glucose-1-phosphate adenylyl transferase	down-regulated	4
*PYG*	glycogen phosphorylase	down-regulated	3
*bam*	beta-amylase	down-regulated	1
*Pgm*	phosphoglucomutase	down-regulated	1
*malQ*	4-alpha-glucanotransferase	down-regulated	2
*GBE1*	1,4-alpha-glucan branching enzyme	down-regulated	3
*INV*	beta-fructofuranosidase	down-regulated	3
*AMY*	alpha-amylase	down-regulated	1
*ALDO*	fructose-bisphosphate aldolase	down-regulated	4
*PGK*	phosphoglycerate kinase	down-regulated	1
*gpmB*	probable phosphoglycerate mutase	down-regulated	1
*PK*	pyruvate kinase	down-regulated	1
*PDHA*	pyruvate dehydrogenase E1 component alpha subunit	down-regulated	1
*ACLY*	ATP citrate (pro-S)-lyase	down-regulated	2
*MDH*	malate dehydrogenase	down-regulated	2
*NdhJ*	NAD(P)H-quinone oxidoreductase subunit J	down-regulated	1
*NdhF*	NAD(P)H-quinone oxidoreductase subunit 5	down-regulated	1
*CYTB*	ubiquinol-cytochrome c reductase cytochrome b subunit	down-regulated	2
*COX1*	cytochrome c oxidase subunit 1	down-regulated	1
*COX2*	cytochrome c oxidase subunit 2	down-regulated	1
*COX3*	cytochrome c oxidase subunit 3	down-regulated	1
*ATPF1G*	F-type H^+^-transporting ATPase subunit gamma	down-regulated	1
*ATPF0B*	F-type H^+^-transporting ATPase subunit b	down-regulated	1
*ATPF1D*	F-type H^+^-transporting ATPase subunit delta	down-regulated	1
*ATP1*	F-type H^+^-transporting ATPase subunit alpha	down-regulated	1
*ATP2*	F-type H^+^-transporting ATPase subunit beta	down-regulated	1
*ATP6*	F-type H^+^-transporting ATPase subunit a	down-regulated	1
*ATP6B*	V-type H^+^-transporting ATPase subunit B	down-regulated	2
*AMY*	alpha-amylase	up-regulated	1
*INV*	beta-fructofuranosidase	up-regulated	1
*TPS*	trehalose 6-phosphate synthase/phosphatase	up-regulated	6
*GPI*	glucose-6-phosphate isomerase	up-regulated	1
*PFK*	6-phosphofructokinase 1	up-regulated	2
*ND4*	NADH-ubiquinone oxidoreductase chain 4	up-regulated	1
Sum			76

## Data Availability

The data presented in this study are available in the figures and tables.

## Abbreviations

ACC:	1-aminocyclopropane-1-carboxylic acid
ACS:	ACC synthase
AMY:	α-amylase
ATP:	Adenosine triphosphate
CAT:	Catalase
CBL:	Calcineurin B-like protein
CDPK:	Calcium/calmodulin-dependent protein kinases
CEL:	Cellulase
CIPK:	CBL interacting protein kinases
CK:	Control
CML:	Calcium-binding proteins and calmodulin-like protein
CO_2_:	Carbon dioxide
CTATP:	Ca^2+^-transporting ATPase
DEGs:	Differentially expressed genes
DS:	Deep submergence
EHDCP:	EH domain-containing protein
EMP:	Embdem–Meyerhof–Parnas
ENO:	Enolase
ERTF:	Ethylene-responsive transcription factor
FBA:	D-fructose-1,6-diphosphate
GAPDH:	Glyceraldehyde-3-phosphate dehydrogenase
GPX:	Glutathione peroxidase
GPI:	Glucose-6-phosphate isomerase
GSR:	Glutathione reductase
GST:	Glutathione s-transferase
H_2_O_2_:	Hydrogen peroxide
HXK:	Hexokinase
INV:	Beta-fructofuranosidase
LHC:	Light-harvesting chlorophyll protein complex
MIOX:	Myo-inositol oxygenase
MT:	Metallothionein
NADPH:	Nicotinamide adenine dinucleotide phosphate
O_2_$\bar{\cdot }$	Superoxide anion radical
PCD:	Programmed cell death
PFK:	Phosphofructokinase
PG:	Polygalacturonase
PGAM:	Phosphoglyceratemutase
PGK:	Phosphoglycerate kinase
PK:	Pyruvate kinase
RBOH:	NADPH oxidase respiratory burst oxidase homolog
ROS:	Reactive oxygen species
SOD:	Superoxide dismutase
SS:	Shallow submergence
TCA cycle:	Tricarboxylic acid cycle
TGDR:	Three Gorges Dam Reservoir
TPI:	Triose-phosphate isomerase
XET:	Xyloglucan endotransglucosylase
